# Utilisation of subsidised genetic and genomic testing in a publicly funded healthcare system 2014–2023

**DOI:** 10.1038/s41431-025-01801-4

**Published:** 2025-02-05

**Authors:** Chris Schilling, Florencia Sjaaf, Ilias Goranitis, Kim Dalziel, Melissa Martyn, Zornitza Stark, Clara Gaff

**Affiliations:** 1 Melbourne Health Economics, University of Melbourne, Melbourne, VIC Australia; 2 Australian Genomics Health Alliance, Melbourne, VIC Australia; 3 Murdoch Children’s Research Institute, Melbourne, VIC Australia; 4 Melbourne Genomics Health Alliance, Walter and Eliza Hall Institute, Melbourne, VIC Australia; 5 Department of Paediatrics, University of Melbourne, Melbourne, VIC Australia; 6 Victorian Clinical Genetics Services, Murdoch Children’s Research Institute, Melbourne, VIC Australia

**Keywords:** Health care economics, Genetic testing

## Abstract

The Australian government subsidises medical services, including several genetic and genomic tests, through a federal funding scheme. We explore trends and variation in the utilisation of the publicly funded genetic and genomic tests over the last decade. We make use of administrative data of the listed genetic and genomic tests from financial year 2014 to 2023. In 2023, 102 genetic and nine genomic tests were publicly subsidised across 65 distinct clinical test indications, up from 32 items across 20 distinct tests in 2014. Service volumes have increased by 50% from 250,881 to 376,140, and benefits paid have risen by 83% from AU$42.0 million to AU$76.8 million. This accounts for 0.3% of the total AU$27.6 billion expenditure on publicly subsidised medical services in 2023. Somatic cancer, rare disease, and reproductive tests are the most prevalent tests. Women of childbearing ages used more services than men, however in nonchildbearing ages, men used more services than women. The current usage of publicly funded genetic and genomic testing within Australia is relatively modest, underscoring challenges in integration to routine clinical practice. However, the recent rapid expansion of subsidised items indicates that investments into genomics research are beginning to yield the evidence necessary to secure public funding for these services.

## Introduction

Genetic and genomic testing is revolutionising the field of medicine by offering more precise diagnoses, prognoses, and treatment strategies [1]. In Australia, the utilisation of several genetic and genomic testing services is subsidised by the federal government through listing the test items on the Medicare Benefits Schedule (MBS) [2]. From this listing, clinicians can order tests for patients, and the latter will be reimbursed some or all of the fee. The earliest listed test on the MBS were chromosome analyses which were mainly used for reproductive, rare disease, and somatic cancer purposes [3, 4]. New items were then listed for genetic testing of specific genes, and genomic testing of multiple genes extending to exome or genome sequencing [5]. Clinical indications have also broadened to more rare diseases, cancer, haematological, and immunological disorders, as well as codependent tests that assess eligibility for subsidised drug treatment [2, 6]. In cancer, listed items have expanded to include both somatic tests of tumour cells and germline tests which can inform inherited predispositions to certain types of cancers [7].

Challenges have arisen alongside these developments [1, 8, 9]. To be listed on the MBS and be government subsidised, a health technology assessment (HTA) must be undertaken that appraises the safety, efficacy, and cost-effectiveness of the technology [6, 10]. For rapidly evolving fields, such as genomics, HTA can be challenging and time-consuming, and require the government to make difficult trade-offs between accepting more uncertainty or delaying adoption of genomics into clinical practice [6]. Current HTA processes are well-designed to evaluate medicines with tightly scoped clinical indications, informed by clinical trials with large sample sizes that assess safety and efficacy [11]. By contrast, genomic testing can exploit differences between individuals to better target clinical management across multiple indications. It has the potential to fundamentally alter disease progression and treatment not just for individuals but also for family members [11]. As a result, it is harder to evaluate.

Establishing data transparency on test performance, including a comprehensive overview of available, utilised, and funded tests, is a key step in assessing current efforts to support the adoption and implementation of genetic and genomic services [12, 13]. Previous Australian studies have shown the underutilisation of rare disease testing in children [14], and the disparities in access to genetic services among the Indigenous communities, especially females and those in remote areas [15]. However, a broader capture of funded genetic and genomic services utilisation remains scarce.

This article explores the dynamic evolution of the publicly funded genetic and genomic testing in Australia over the last ten years, highlighting key trends in the number and type of items listed on the MBS, the volume of services delivered and the value of government expenditure outlaid. We also investigate the equity of access to services across Australia’s states and territories over time. Finally, we report on the distribution of services and funding by age and sex, to better understand who currently benefits the most from genetic and genomic testing. By reviewing the journey thus far, we can better anticipate the opportunities and challenges that lie ahead for genetic and genomic testing and its integration into mainstream medical practice.

## Methods

We obtained a decade of administrative MBS data for the P7 Genetics group of Category 6 Pathology services via Medicare Statistics Services Australia on 16^th^ August 2023. The P7 Genetics group includes both genetic and genomic tests. Medicare Statistics Services Australia reports service use and benefits paid both as counts and as per capita figures, by patient location, age and sex [16]. These data were analysed to assess linear and quadratic time trends for financial years 2014–2023, and to provide a range of metrics, first at the national level, and then disaggregated by state, age and sex. Tests were classified into type (genetic or genomic), categories (somatic cancer, germline cancer, rare disease, reproductive, haematological, immunological, or pharmacogenomic), and uniqueness (distinct, cascade, or other similar items) by a clinical geneticist (ZS). The latter classification was made to identify tests covering distinct clinical indications as the MBS can list multiple items for the same tests, for example, separate items for testing an individual versus a family member, known as cascade tests [17]. Definitions and classifications of each item are available in the Supplementary Table S2. Benefits paid were analysed in nominal unadjusted terms as reported by Medicare Statistics Services Australia, noting that there is typically no indexation for these items. These reported amounts reflect the government’s contribution. Total amounts paid for the services, including patient contributions, were not captured in the provided dataset. The items’ descriptions, including their starting date and MBS rebate, were collected from the MBS Online website [2]. The descriptions for items that were no longer listed on the MBS in 2023 were collected from their latest Medicare benefits schedule book’s description [18]. Items that were listed on the MBS but had zero utilisation were excluded. All analyses were completed in Microsoft Excel and Stata version 17 (StataCorp LLC).

## Results

### Number of listed items, service volumes and benefits paid

Over the last decade, the number of genetic and genomic testing items listed on the MBS has more than tripled from 32 in 2014 to 111 in 2023 (Table 1). The number of distinct items, after excluding cascade and other similar items, has also tripled, from 20 in 2014 to 65 in 2023. This has been accompanied by a 50% increase in service volumes over the same period, rising from 250,881 in 2014 to 376,140 in 2023. The benefits paid reached AU$76.8 million in 2023, comprising 0.3% of the total AU$27.6 billion MBS expenditure on all publicly subsidised medical services during the year. This is up 83% from the AU$42.0 million genetic and genomic testing benefits paid in 2014. Relative to the growth of total MBS expenditure (4.1% per annum), the expenditure for genetic and genomic testing have grown at 6.9% over the last decade.

**Table 1:** Publicly subsidised test numbers (total and distinct), service volumes, and benefits paid, financial years 2014–2023.

Financial year	Number of distinct listed items	Number of total listed items	Service volumes	Benefits paid
2014	20	32	250,881	AU$42,020,322
2015	20	33	260,696	AU$41,770,639
2016	20	34	264,251	AU$43,625,187
2017	21	33	280,912	AU$45,657,085
2018	23	38	318,679	AU$50,392,939
2019	26	46	328,506	AU$53,177,962
2020	40	63	326,284	AU$54,688,860
2021	46	76	371,507	AU$65,486,155
2022	53	90	338,648	AU$64,640,665
2023	65	111	376,140	AU$76,832,739
CAGR	14.0%	14.8%	4.6%	6.9%
Linear trend coefficient	5.01*** (0.8281)	8.59*** (0.8310)	14,274*** (0.8972)	AU$3,712,649*** (0.8979)
Quadratic trend (adj R-squared)	0.47*** (0.9562)	0.81*** (0.9634)	1,216*** (0.8205)	AU$339,874*** (0.9671)

*CAGR* compound annual growth rate.

***p-value < 0.001.

**Fig. 1:**
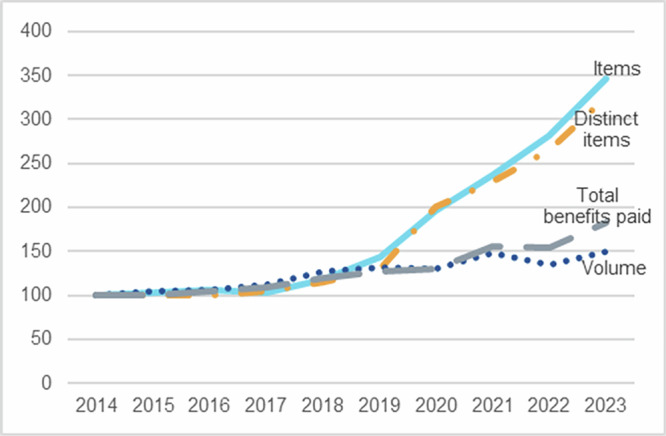
Index of number of listed item (total and distinct), service volumes and benefits paid, financial years 2014–2023, national. Growth in listed item numbers has accelerated in the last four years, outstripping growth in service volumes and benefits paid. (Index 2014 = 100).

While the annual growth rates from 2014 to 2023 for total listed items and distinct items are 14.8% and 14.0%, respectively, most of this growth has occurred over the last four years. Since 2020, 65 new items or 39 new distinct items have been added and utilised. This is reflected in the rise in CAGR for total items, which increased from 7.5% from 2014 to 2019 to 20.8% from 2020 to 2023. Similarly, the CAGR for total distinct items increased from 5.4% to 17.6% over the same period. This is reflected in the statistically significant quadratic time trends and model fit (Table 1). By contrast, services and value of benefits paid have not grown at the same rate over that period (Fig. 1). The value of benefits paid has increased at a faster rate than service volumes reflecting the addition higher cost items.

Most listed items are genetic tests, with only nine genomic tests listed in 2023. Somatic cancer, rare disease, and reproductive tests are the dominant categories in all metrics. Overall, growth in services has been relatively consistent across all categories, except for germline cancer tests which have grown at a faster rate, albeit from a low base (Supplementary Table S1).

### High volume items

The two most commonly utilised items in 2023 were the ‘study of the whole of every chromosome by cytogenetics or other techniques’ (item 73289) in the reproductive test category and a haematological test of detection of common haemochromatosis variants (item 73317), which combined make up over 130,941 services or a third of 2023 volumes. These genetic test items have been listed for over a decade and have experienced only modest volume annual growth of 4.5% and 1.0% respectively. By contrast, the next most common items in 2023, detection of HLA-B27 (item 73320) in the immunological category and gene rearrangements for the diagnosis and monitoring of certain types of leukemia (item 73314) in the somatic cancer category, have experienced annual growth over the last decade of 17.7% and 11.6% respectively. Most new items listed over the last four years, including all genomic tests, have relatively low volumes to date. The services utilisation and benefits paid per capita in each item category are available in Supplementary Fig. S3.

### High-cost items

**Fig. 2:**
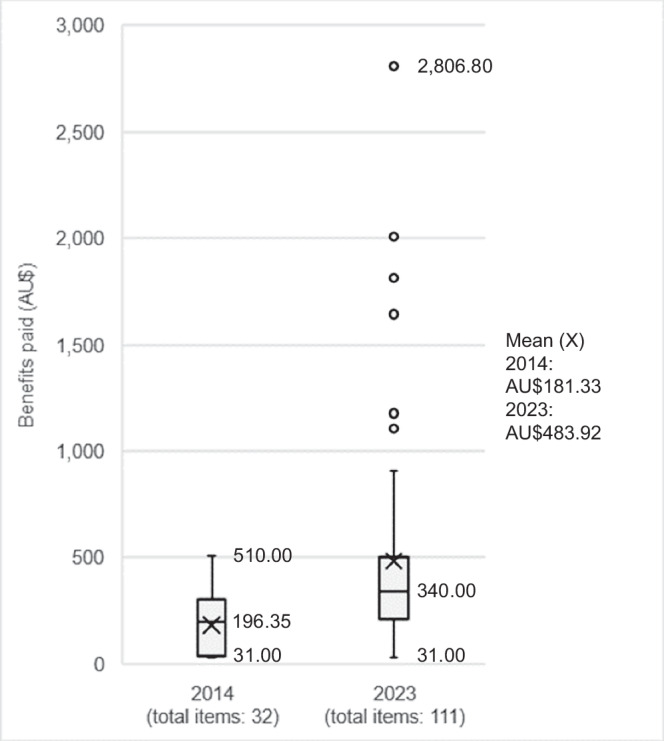
Government rebates of listed items in 2014 and 2023. Median rebates have increased markedly since 2014. Newer items have a higher rebate.

There are five items in 2023 with MBS rebates higher than AU$1,800: these are exome and genome sequencing items for syndromic and non-syndromic intellectual disability in children aged 10 years or younger (items 73358 and 73359) and heritable kidney disease (73401 and 73402); and an embryo screening service for assisted reproductive technology (73387). These are relatively new items listed on or after May 2020, with low volumes (Table 2). Most of the high-cost items fall under the rare disease and reproductive test category (Supplementary Fig. S1). While item 73387 is a genetic test from the reproductive category, the other four items are genomic tests under the rare disease category. The median reimbursement for items listed in 2023 is AU$340 (range from AU$31 to AU$2,806.80), up from AU$196 (AU$31-510) in 2014 (Fig. 2).

**Table 2:** Top five most expensive government rebates for genetic and genomic testing items, financial years 2014 and 2023.

Item number	Description	Category	Type	Government rebate	Service volumes	Benefits paid	Year introduced
2014							
73333	Detection of germline mutations of the von Hippel-Lindau gene	Germline cancer	Genetic	AU$510.00	28	AU$14,820	2013
73292	Chromosomes analysis by genome-wide micro-array for developmental delay, intellectual disability, autism, or at least two congenital abnormalities	Rare disease	Genetic	AU$501.45	20,948	AU$10,594,644	2010
73328	Test of tumour tissue in patient with locally advanced or metastatic non-small cell lung cancer, related to EGFR gene for accessing gefitinib under federal funding scheme	Somatic cancer	Genetic	AU$337.75	12	AU$3855	2012
73337	Test of tumour tissue in patient with non-small cell lung cancer, related to EGFR gene status for accessing EGFR tyrosine kinase inhibitor or immunotherapy under federal funding scheme	Somatic cancer	Genetic	AU$337.75	5964	AU$1,945,557	2022
73287	Chromosome analysis by cytogenic or other techniques on one or more tissue or fluid except blood–one or more tests	Reproductive	Genetic	AU$335.40	9227	AU$2,986,330	1994
2023							
73359	Whole exome or genome sequencing and analysis for syndromic and non-syndromic intellectual disability in children, once a lifetime, includes samples from parents	Rare disease	Genomic	AU$2806.80	707	AU$1,986,562	2020
73358	Whole exome or genome sequencing and analysis for syndromic and non-syndromic intellectual disability in children, once a lifetime	Rare disease	Genomic	AU$2006.80	144	AU$289,587	2020
73401	Whole exome or genome sequencing and analysis of germline variants for heritable cystic kidney disease	Rare disease	Genomic	AU$2006.80	102	AU$204,900	2023
73402	Whole exome or genome sequencing and analysis, of germline variants for heritable kidney disease except cystic or Alport syndrome	Rare disease	Genomic	AU$2006.80	163	AU$327,416	2023
73387	Genetic analysis of embryonic tissue from 3 or more embryos from assisted reproductive treatment, for pre-implantation genetic test	Reproductive	Genetic	AU$1811.80	554	AU$1,292,728	2022

*EGFR* epidermal growth factor receptor.

### Variation by state

New South Wales, Queensland, the Australian Capital Territory and Tasmania all have services per capita slightly above the national average in 2023, while Victoria and South Australia have services per capita at 98% of the national average (Supplementary Fig. S2). New South Wales and Victoria have benefits paid per capita 14% and 5% higher than the national average, respectively.

Northern Territory consistently ranks last in all the per capita metrics for 2023 and across the last decade. Services per capita and benefits paid per capita remained at just over half the national average across the decade. Western Australia ranks second-last behind the Northern Territory in services per capita and benefits paid per capita, at 69% and 61% of the national 2023 average, respectively. The services and benefits paid per capita of each test category in each state are available in Supplementary Fig. S4.

### Variation by demographics

**Fig. 3:**
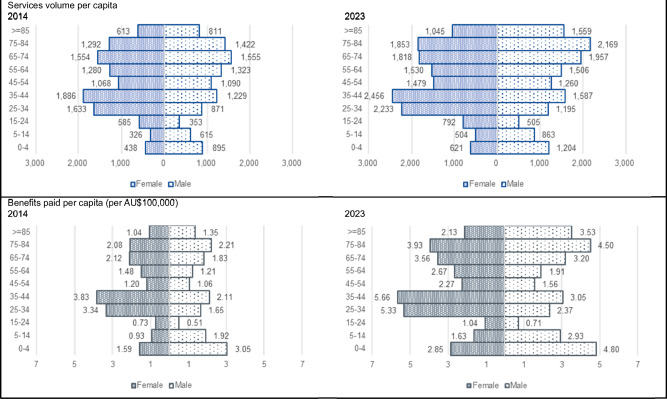
Service volumes per capita and benefits paid per capita, 2014 and 2023, by age and sex. Services and benefits paid per capita vary across the life course.

Services per capita and benefits paid per capita vary by age and sex (Fig. 3). By age, children and young adults under 25 have markedly lower services per capita and benefits paid per capita than older people. By sex, women in childbearing ages (aged 15 to 44 years) have higher benefits paid per capita than similarly aged males, reflecting the range of items associated with pregnancy screening. In early childhood, and the later years, males have higher levels of benefits paid. All age and sex cohorts have experienced relatively similar changes in usage and funding over the last decade.

## Discussion

Current federal funding of genetic and genomic tests is modest at AU$76.8 million, or less than 0.3% of the total AU$27.6 billion total expenditure on publicly subsidised medical services during 2023 [16]. Benefits paid have grown at 6.9% per annum over the last decade, faster than total publicly subsidised medical services expenditure (4.1%), but perhaps lower than might be expected given the level of technological advancement and investment in translational genomics research. Within the sector, the uptake of genetic and genomic testing in mainstream Australian clinical practice is sometimes referred to as ‘slow’ [19]. While ‘slow’ is subjective, our descriptive analysis perhaps supports this assertion for volumes and benefits paid, but not for the number of items listed on the MBS which has more than tripled in the last decade. This is true even after excluding non-distinct items such as cascade tests.

While the extensive processes involved in gaining federal funding can be a barrier to timely adoption of genetic and genomic testing [8], the growth in the number of items listed on the MBS over the last four years in particular suggests that the funding of clinical and health economics research is delivering the evidence-base to support successful HTA.

However, there is still work to be done. While new items have been listed, current uptake is low, and lower than predicted [14]. Challenges to increased adoption of genetic and genomic testing include the capability and capacity of the workforce, and a lack of sustainable service models. With the increase in availability of tests must come an increase in the availability of the services which includes workforce education and support to ensure patient access and appropriate use [20]. Currently, many non-genetic medical specialists would rather refer to genetic specialists for genomic testing [21], making some genomic testing less accessible or timely outside of central tertiary centres. Education and training have been shown to be effective at improving clinician confidence and understanding of genomic medicine [22]. Similarly, further efforts in implementation science are required to develop and embed the spectrum of sustainable service models necessary for mainstream adoption of genetic care [1, 19, 23]. Another opportunity for improvement is in the use of genomic testing with only nine items currently listed and annual benefits paid at just AU$3.3 million. This compares with the 251 clinical indications which have a genomic test funded by the National Health Service England [24].

Our study shows substantial geographic variation in the provision and funding of genetic testing across Australia. This has previously been observed for individual listed items for specific diseases [14], but our study highlights the variation at the aggregate level. The results paint a particularly concerning picture for the Northern Territory, the region with the most regionally dispersed population and the highest share of Indigenous peoples, whose access and funding remains at around half that of the national average. There are multiple potential causes for the observed variation, including different resourcing models for testing that rely less on the federal funding, disparate levels of investment in infrastructure, and variation in workforce capacity and capability across Australia states [14]. For example, the Northern Territory Department of Health contracts the Victorian Clinical Genetics Services to provide genetic services in clinics in two of the Northern Territory cities. Genetic specialists based in Victoria visit the clinics approximately four times each year and provide telehealth appointment when suitable [15, 25]. These tests are not captured in the MBS data presented here, but even with such tests, patients still face greater barriers in accessing services compared to other states with localised services. Collation of data across funding models is critically required to better understand the level of resourcing in the Northern Territory.

Our study also observed demographic variation in the provision and funding of genetic testing across Australia. While the variation tends to follow expected disease and testing norms, there are some interesting observations. First, despite the potential lifetime benefits of genomic testing, currently children and young adults have markedly lower use rates per capita than older persons. This is partially explained by the type of tests currently listed and federally-funded, which are typically for diseases that manifest later in life. Second, while women on average have higher use rates of genetic testing than men, the majority of men outside the of 15–44 age group have higher usage rates than women. In early childhood 0–4 years of age, expenditure on genetic testing is double for young boys than young girls, partially explained by higher use of intellectual disability tests, including for Fragile X syndrome which disproportionately affects boys [26]. In older age, men tend to utilise somatic cancer tests category more than women. This, in addition to the haemochromatosis test, push up the service volumes and benefits paid for men. This is not entirely expected given that women traditionally access health services at a markedly higher rate than men. It is a timely reminder that increasing uptake of genomic services will require gender-specific targeting of health services, because men and women think and act differently about health and help seeking [27, 28].

A limitation of this research is that it provides only observed data, and cannot make normative judgements on the optimal diffusion and funding of genetic and genomic tests. For example, it may be optimal that there is less rather than more testing, if a single one-off genomic test can replace multiple tests over many years. A second limitation is data coverage. The provision of genetic and genomic testing in Australia is complex, including both federal and state government funding arrangements, service provision between public and private health services, and other non-MBS funded tests that consumers pay for themselves [8]. Due to the lack of a common data frame, our study has not investigated trends in the volume and funding of non-MBS funded tests.

As we reflect on the progress made in the field of publicly subsidised genetic and genomic testing in Australia, we also consider the future trajectory in translating genetic and genomic medicine into clinical services. Rapid developments in technologies, coupled with the growing availability of large-scale genomic databases, hold the promise of more precise risk assessments, earlier disease detection, and targeted therapeutic interventions. As seen from our findings in Australia landscape, the trends in usage and funding to date are relatively modest, but encouragingly investments in genomic research are translating to more subsidised tests available for patients.

## Supplementary information

Supplementary Figures

Supplementary Table S1

Supplementary Table S2

## Data Availability

Data is readily available from Medicare Statistics and MBS Online website for utilisation in aggregated form.
