# Supplementary material for: Utilisation of subsidised genetic and genomic testing in a publicly funded healthcare system 2014–2023
Source: Eur J Hum Genet. 2025 Feb 5;33(8):1044–50. doi: 10.1038/s41431-025-01801-4 (PMC12322065; doi:10.1038/s41431-025-01801-4)
Supplement: Supplementary file 1 — Supplementary Figures [file 41431_2025_1801_MOESM1_ESM.pdf]

Supplementary materials

Utilisation of subsidised genetic and genomic testing in a publicly funded healthcare system 2014-2023

Chris Schilling[1], Florencia Sjaaf[1], Ilias Goranitis[1,2,3], Kim Dalziel[1,3], Melissa Martyn[3,4], Zornitza Stark[2,5,6], Clara Gaff[3,4,6]

[1] Health Economics Unit, Centre for Health Policy, University of Melbourne, Melbourne, Australia

[2] Australian Genomics Health Alliance, Melbourne, Australia

[3] Murdoch Children's Research Institute, Melbourne, Australia

[4] Melbourne Genomics Health Alliance, Walter and Eliza Hall Institute, Melbourne, Australia

[5] Victorian Clinical Genetics Services, Murdoch Children's Research Institute, Melbourne, Australia

[6] Department of Paediatrics, University of Melbourne, Melbourne, Australia

Contents:

**Supplementary Figure S1**: MBS rebates from lowest to highest per category in 2023. The high-cost items belong to the rare disease and reproductive tests category	2
**Supplementary Figure S2**: Service volumes per capita and benefits paid per capita, 2014 and 2023, by state.	3
**Supplementary Figure S3**: Service volumes and benefits paid per capita of each test category in 2014 and 2023.	4
**Supplementary Figure S4**: Service volumes and benefits paid per capita of each test category in 2014 and 2023, by state.	4

Supplementary Figure S1: MBS rebates from lowest to highest, per category, in 2023. The high-cost items belong to the rare disease and reproductive tests category.

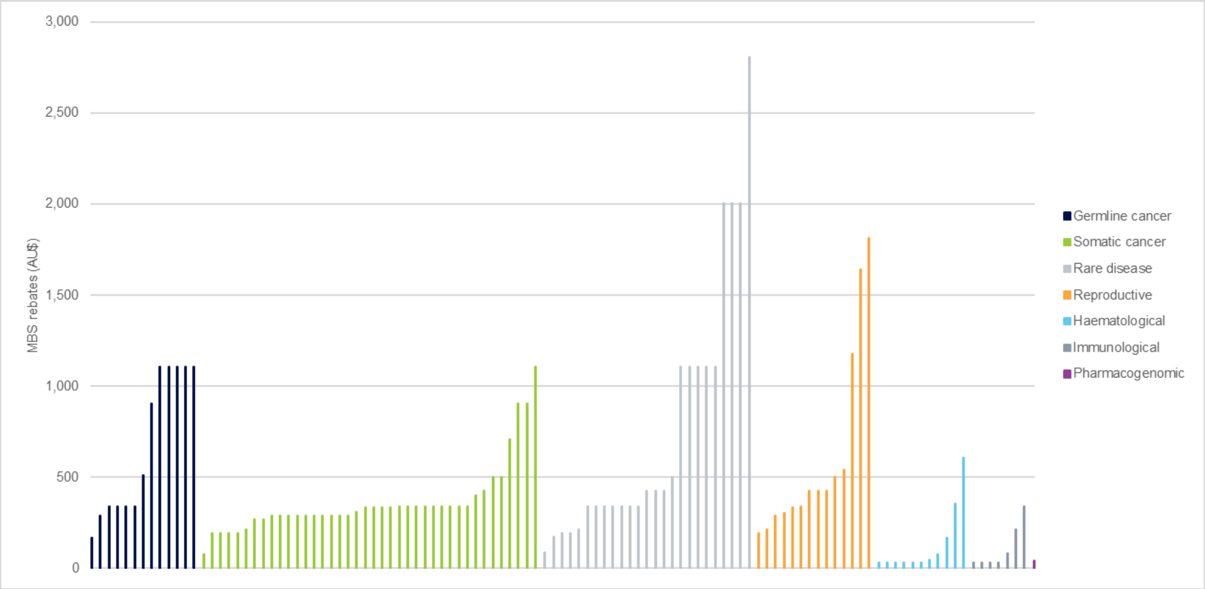

MBS: Medicare Benefits Schedule.

Supplementary Figure S2: Service volumes per capita and benefits paid per capita, 2014 and 2023, by state. Services and benefits paid per capita in the Northern Territory have remained at just over half over the national average across the last decade.

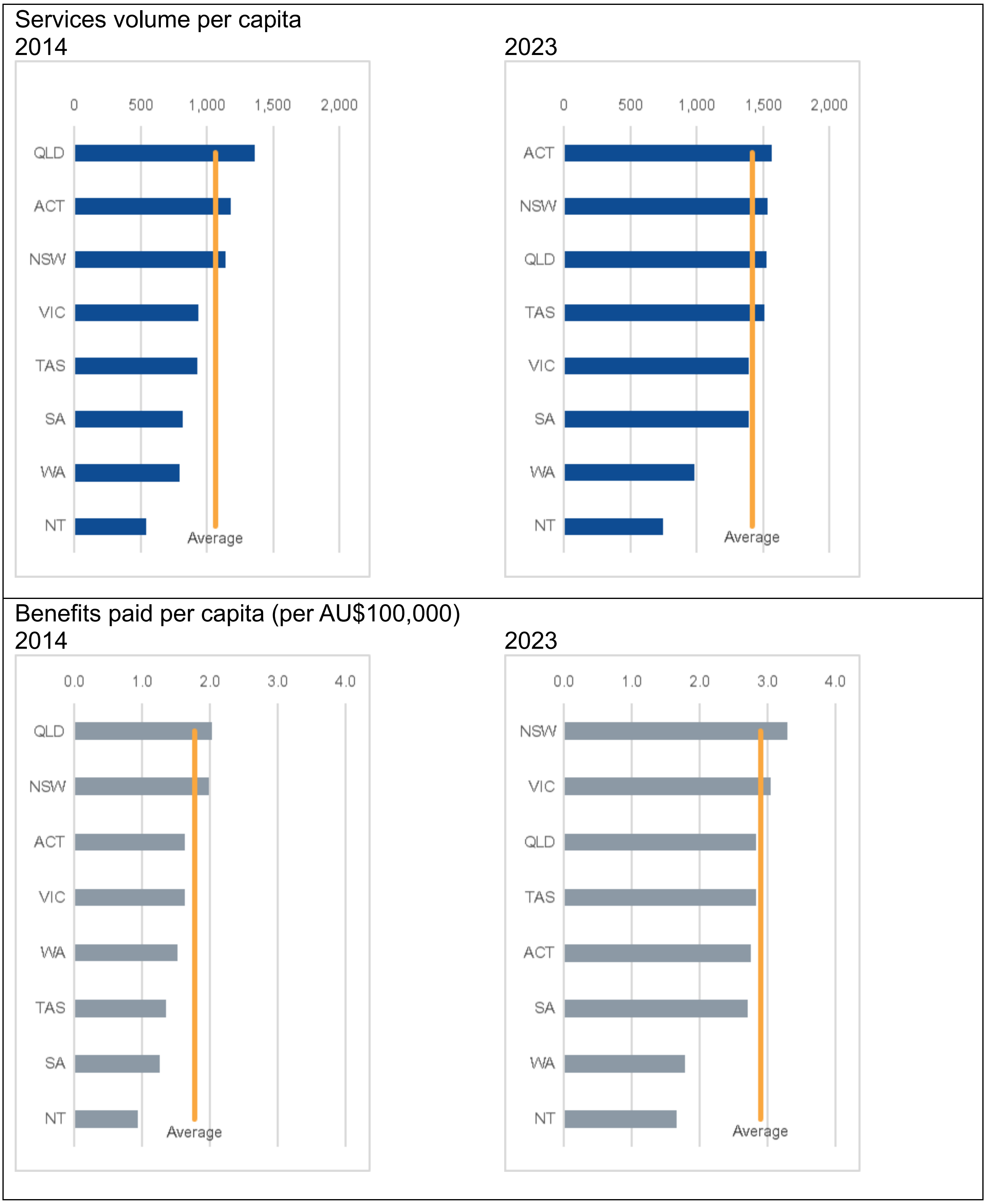

NSW: New South Wales; VIC: Victoria; QLD: Queensland; SA: South Australia; WA: Western Australia; TAS: Tasmania; ACT: Australian Capital Territory; NT: Northern Territory.

Supplementary Figure S3: Service volumes and benefits paid per capita of each test category in 2014 and 2023.

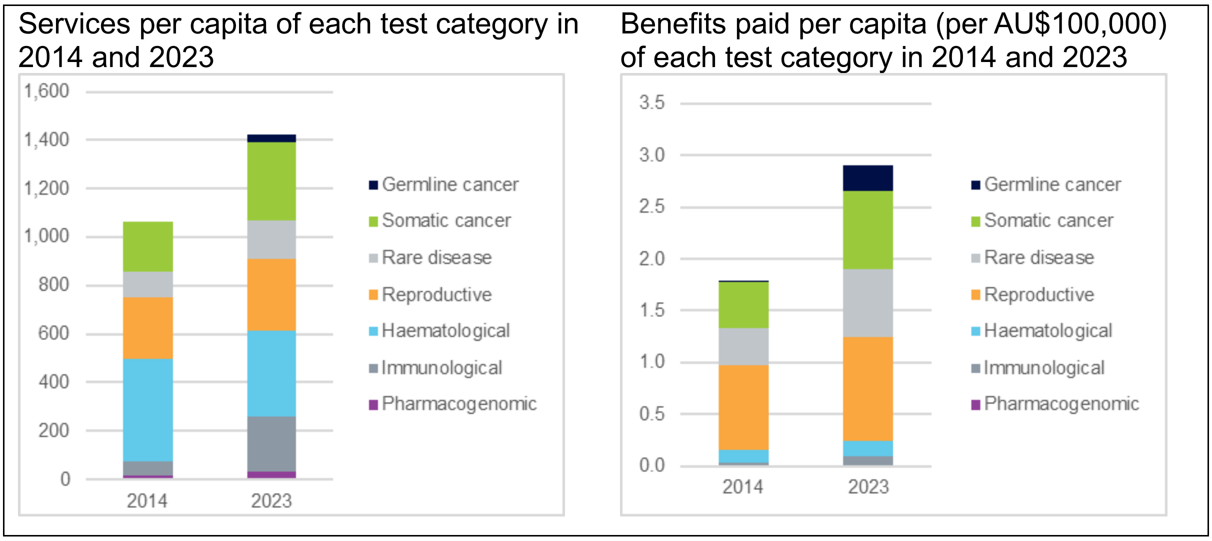

Supplementary Figure S4: Service volumes and benefits paid per capita of each test category in 2014 and 2023, by state.

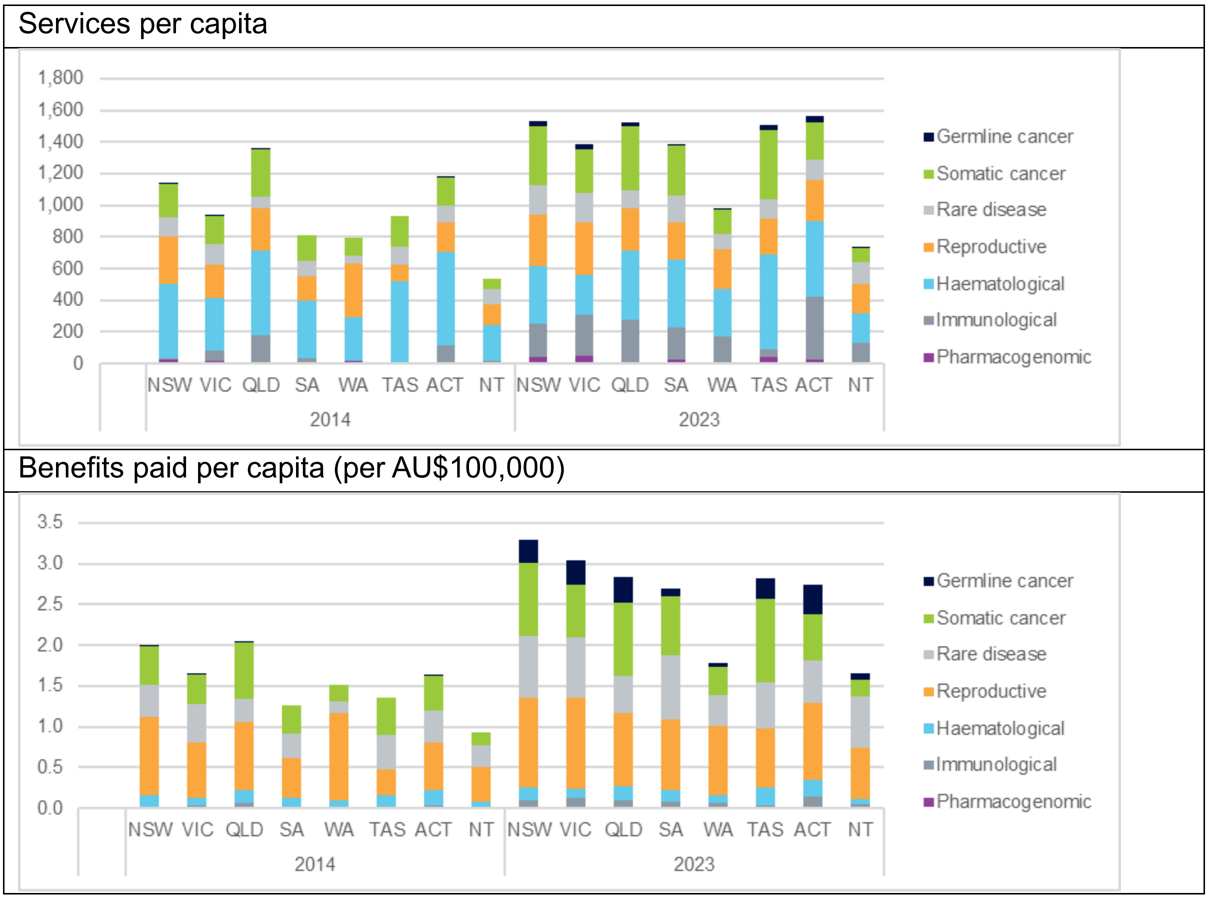

NSW: New South Wales; VIC: Victoria; QLD: Queensland; SA: South Australia; WA: Western Australia; TAS: Tasmania; ACT: Australian Capital Territory; NT: Northern Territory.
